# Not all inequalities are equal: differences in coverage across the continuum of reproductive health services

**DOI:** 10.1136/bmjgh-2019-001695

**Published:** 2019-09-03

**Authors:** Elizabeth A Sully, Ann Biddlecom, Jacqueline E Darroch

**Affiliations:** Guttmacher Institute, New York City, New York, USA

**Keywords:** Reproductive health services, maternal health services, contraception, universal coverage

## Abstract

Reducing inequalities in health service coverage is central to achieving the larger goal of universal health coverage. Reproductive health services are part of evidence-based health interventions that comprise a minimum set of essential health interventions that all countries should be able to provide. This paper shows patterns in inequalities in three essential reproductive health services that span a continuum of care—contraceptive use, antenatal care during pregnancy and delivery at a health facility. We highlight coverage gaps and their impacts across geographical regions, key population subgroups and measures of inequality. We focus on reproductive age women (15–49 years) in 10 geographical regions in Africa, Asia and Latin America and the Caribbean. We examine inequalities by age (15–19, 20–24, 25–34 and 35–49 years), household wealth quintile, residence (rural or urban) and parity. Data on service coverage and the population in need are from 84 nationally representative surveys. Our results show that dominant inequalities in contraceptive coverage are varied, and include large disparities and impact by age group, compared with maternal health services, where inequalities are largest by economic status and urban–rural residence. Using multiple measures of inequality (relative, absolute and population impact) not only helps to show if there are consistent patterns in inequalities but also whether few or many different approaches are needed to reduce these inequalities and where resources could be prioritised to reach the largest number of people in need.

**Summary box**

- A key part of achieving 100% coverage of health services for people who need them is to focus resources on people who are more disadvantaged and have lower levels of coverage.
- The size and impact of inequalities in service coverage among women in developing regions differ by type of reproductive health service. Age inequalities are common in contraceptive coverage, with adolescent women at a disadvantage, although other inequalities (by parity, wealth and residence) are also predominant.
- Inequalities by household wealth and urban–rural residence are the most pronounced in maternal health service coverage (antenatal care and delivery in a health facility), with poorer women and women living in rural areas at greater disadvantage than other women.
- Programme and policy efforts to improve reproductive health service coverage must recognise the different magnitude and impact of inequalities across services to prioritise resources and approaches better to achieve universal health coverage.

## Introduction

A foundation of national and international commitments to universal health coverage and to the larger development agenda to 2030 under the Sustainable Development Goals (SDGs) is to ensure that all people in need of essential health services receive them.^1 2^ Monitoring inequalities in health service coverage and focusing public and private sector efforts to reduce these inequalities are thus central to achieving the larger goal of universal coverage.^3 4^ Reproductive health services are part of evidence-based health interventions that comprise a minimum set of essential health interventions that all countries should be able to provide.^5 6^ We examine patterns in inequalities in three essential reproductive health services that span a continuum of care—contraceptive use, antenatal care (ANC) during pregnancy and delivery at a health facility—to assess coverage gaps and their impacts across geographical regions, key population subgroups and measures of inequality.

Multi-country studies on health coverage inequalities tend to focus on a particular intervention or population subgroup.^7–11^ Without comparisons across interventions and subgroups, we miss a broader understanding of regularities in inequalities in health coverage and if similar approaches can be taken to reduce inequalities.^12^ Moreover, common measures of inequality that compare two groups (eg, urban vs rural residents; people in the poorest households vs the richest households) are straightforward to interpret but leave out information for subgroups with more than two categories as well as the size of the populations affected.^3^ Such information also leads to a more complete understanding of how inequalities are patterned and the resources needed to improve coverage.^9^

Estimates for this analysis come from the Adding it Up study,^13^ which is an analysis of the need and coverage and cost and benefits of contraceptive and maternal and newborn care in developing regions. Data are from 84 nationally representative surveys, from which data on need for and use of contraception, ANC and delivery services were available across sociodemographic categories of age, parity, residence and household wealth (online supplementary table 1). The surveys include those from multi-country programmes such as the Demographic and Health Surveys and the Multiple Indicator Cluster Surveys as well as other nationally representative surveys. The most recent survey estimates were used and applied to 2017 populations to set a uniform reference year for the analysis.^13^

10.1136/bmjgh-2019-001695.supp1 Supplementary data

Our analysis covers reproductive age women (15–49 years) in Africa, Asia and Latin America and the Caribbean. We generate estimates at the regional level to provide a succinct and overarching picture of health service coverage inequalities in parts of the world where gaps in coverage tend to be the greatest. We used country-level data weighted by the country’s relevant population size in 2017 (women of reproductive age or live births) to generate regional estimates. We limited analysis to geographical regions where survey tabulations covering all subgroups were available for at least 50% of women aged 15–49 and of recent births. We thus excluded four regions from analysis (Southern Africa, Eastern Asia, Central Asia and Oceania; online supplementary table 2). We estimated contraceptive need and coverage for never-married women in most countries in North Africa, Southern Asia, Southeast Asia and Western Asia because survey information was not available for them though it was available for ever-married women in the country. Never-married women were estimated to account for less than 2% of women in need of modern contraception in these regions.

The three specific indicators of essential reproductive health services we examine are the proportion of women wanting to avoid a pregnancy who are using a modern contraceptive method, the proportion of live births that received four or more ANC visits, and the proportion of live births delivered in a health facility. These are not comprehensive indicators of contraceptive and maternal and newborn healthcare, but instead represent entry points to care and a minimum standard. Full coverage for each indicator would be 100%.

Women are classified as wanting to avoid a pregnancy and in need of modern contraceptives if they or their partner are currently using a contraceptive method, either traditional or modern; they are currently married or are unmarried and sexually active in the past 3 months, and they are able to become pregnant, and do not want to have a child in the next 2 years; or they identify their current pregnancy as unintended or are experiencing postpartum amenorrhoea after an unintended pregnancy. The measure we use of the proportion of women who want to avoid pregnancy who are using modern contraception is similar to an SDG indicator of the proportion of women of reproductive age who have their need for family planning satisfied with modern methods. Modern contraception includes female and male sterilisation, hormonal methods, intrauterine devices, male and female condoms, modern fertility-awareness-based methods, the lactational amenorrhoea method, emergency contraception and other supply methods. The number of ANC visits and facility delivery are measured from women’s self-reported care received for their most recent birth in the 3 years prior to the survey.

Which groups of women experience the greatest inequalities? We examine four key sociodemographic subgroups: age (15–19, 20–24, 25–34 and 35–49 years), household wealth quintiles, residence (rural or urban) and parity (a two-category subgroup of the number of births a woman has had and where the reference group varies by the outcome of interest). These subgroups were chosen based on findings from previous literature and on conceptual grounds.^14^ Age group estimates show service coverage across the reproductive life course and particularly highlight coverage among adolescents, who often experience social barriers to needed reproductive health services. Household wealth, measured on a relative basis using quintiles, is a proxy for understanding how access to resources may shape service coverage. Rural or urban residence, likewise, may reflect women’s access to services. Finally, parity may differentially impact women’s preferences for and use of contraceptive methods, and their previous experiences with childbearing may affect their use of maternal and newborn health services in their subsequent pregnancies.

## Multiple measures of inequality

How large are the inequalities? We use three measures of inequality. The first two measures reflect the magnitude of differences in coverage levels via a relative measure (the ratio of high to low relative difference in a subgroup) and an absolute measure (the average, absolute mean difference of subgroup categories from the highest level in a subgroup). Both of these measures use unweighted data on service coverage (ie, not accounting for the population size in each subgroup category).

The ratio of the highest level to the lowest level of coverage is an unweighted relative measure of inequality that provides a pairwise comparison of the relative difference in service coverage levels. It does not take into account absolute levels of difference. It is also a simple measure in that it only compares the highest and lowest levels in a subgroup, and thus misses information for subgroups with more than two categories (eg, age groups or household wealth quintiles). Equation 1 shows how the ratio of high to low ($R^{hl}$) is constructed, where *r* is the proportion of women or live births receiving the highest or the lowest level of service coverage within a subgroup.

(1) $$R^{hl}=\frac{r^{h}}{r^{l}}$$

The absolute mean difference from the highest level in the subgroup measures the average difference in service coverage between each category in a subgroup and the category in that subgroup with the highest level of service coverage. The highest level of coverage in the subgroup was selected as the reference group, rather than the overall mean, as a goal of expanding service coverage is to reduce inequalities by bringing everyone up to a highest possible level of coverage. Two key benefits of this measure are that it captures differences across all categories in a subgroup, not just the high and low extremes, and it expresses those inequalities in terms of the absolute size of disparities (for the three coverage indicators, this would be the average percentage point difference from the highest level of coverage in the subgroup). Equation 2 shows how ${MD}^{uw}$ is calculated, where $r^{i}$ is the coverage level for population *i* within a subgroup, and $r^{b}$ is the coverage level of the population with the highest proportion of coverage.

(2) $$MD^{uw}=\sum_{i-1}\frac{r^{i}-r^{b}}{i-1}$$

Finally, we examine the impact of inequalities by measuring how many additional women or births would receive services if women in each subgroup category had the coverage level of the best-covered women in the subgroup. This is calculated by applying the service coverage level from the highest category to the number of women in need of services and subtracting the estimated number of women in that subgroup who currently receive services. For example, if all women had the same level of service coverage as the richest women, how many additional women would (1) be using a modern method of contraception, (2) receive four or more ANC visits and (3) deliver in a health facility? Impact is therefore a weighted measure, where the weights are either the number of women wanting to avoid a pregnancy or the number of women giving birth. This measure allows us not only to assess where inequalities are high, but the subgroups where the largest numbers of women are impacted by inequalities.

## Do inequalities in coverage differ across reproductive health services?

**Figure 1:**
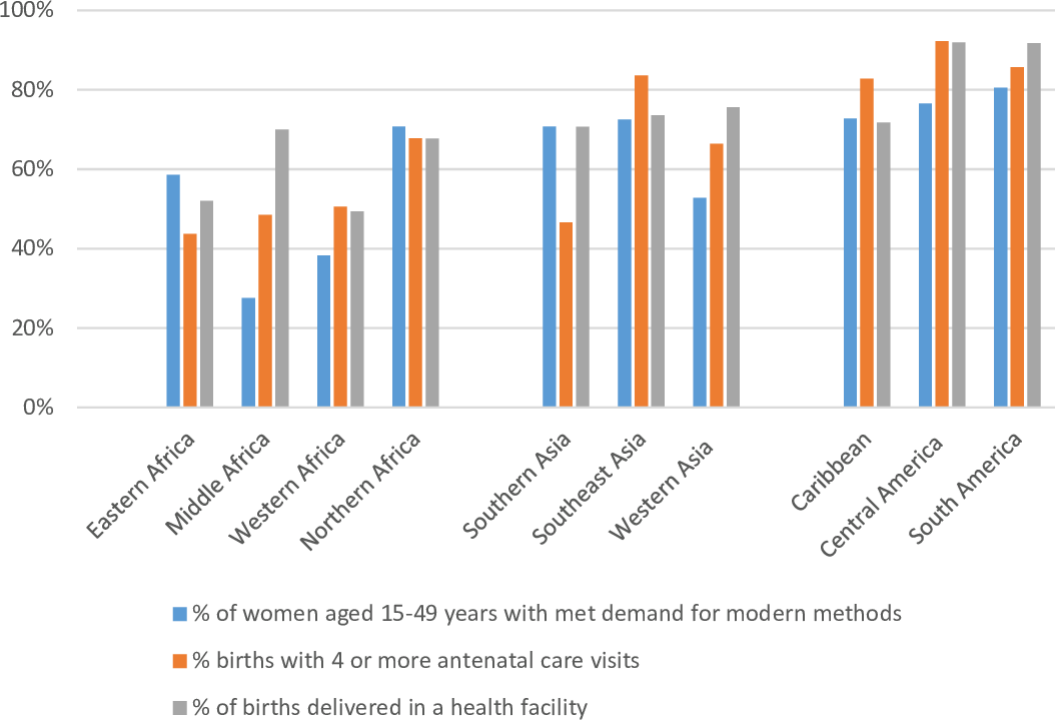
Percentage of relevant population in need covered by contraceptive and maternal health services by region, 2017. Estimates included in the online supplementary table 3.

In 2017, substantial gaps still persisted across developing regions in overall levels of coverage of essential reproductive health services. The level of demand satisfied for modern contraceptive methods ranged from less than 40% in Middle and Western Africa to a high of 81% in South America (figure 1, online supplementary table 3). Coverage tended to be higher for the two maternal health indicators. Coverage of four or more ANC visits ranged from 44% of recent births in Eastern Africa to more than 80% in all regions of Latin America and the Caribbean and in Southeast Asia. Levels of delivery at a health facility ranged from 49% in Western Africa to 70% or higher in regions of Asia and Latin America and the Caribbean (as well as in Middle Africa).

These overall levels of coverage mask widespread inequalities in coverage. In meeting contraceptive needs, ratios of the highest- to lowest-covered subgroup categories show that the largest relative gaps in coverage within regions are varied, with age disparities dominating in five regions, followed by parity (three regions) and household wealth (two regions) (highlighted cells in table 1). The lowest level of coverage tends to be among adolescents (age 15–19 years), women in the poorest household wealth quintile and women who have not started childbearing (online supplementary table 3). The picture of inequalities changes once we look at the indicator of average absolute differences in coverage. Gaps in contraceptive coverage are largest by parity (six regions), ranging from a difference of 12 percentage points in Eastern Africa up to 39 percentage points in Northern Africa. Large age and wealth disparities persist for both relative and absolute measures of inequality in three regions, and residence becomes the largest source of inequality in two regions with coverage that favours urban residents by 12 percentage points (Eastern Africa) and 18 percentage points (Middle Africa). Thus, the average relative inequalities by age are diminished once the smaller differences with other age groups are taken into account. The last inequality indicator accounts for population size within subgroups, and again the picture of inequality in contraceptive service coverage shifts. By raising all women in need to the highest level of coverage in a subgroup, the largest number of women affected within regions are by reducing age group inequalities (five regions), economic status (three regions), residence (Eastern Africa) and parity (Middle Africa).

**Table 1:** Inequality in the proportion of women aged 15–49 years who have their need for family planning satisfied with modern methods by indicator, region and subgroup, 2017

	Relative measure (unweighted pairwise ratio)				Absolute measure (unweighted average absolute mean difference from highest subgroup category)				Population impact measure (number of additional modern contraceptive users if all were like the highest subgroup category (000 s))			
	Age	Wealth	Residence	Parity	Age	Wealth	Residence	Parity	Age	Wealth	Residence	Parity
**Africa**												
Eastern Africa	1.51	1.29	1.21	1.26	0.10	0.09	0.12	0.12	2065	2897	3474	570
Middle Africa	1.19	2.53	1.96	1.68	0.06	0.14	0.18	0.17	570	1250	1197	1722
Western Africa	1.24	2.31	1.53	1.39	0.04	0.20	0.16	0.14	593	3965	2473	3240
Northern Africa	1.84	1.06	1.04	2.19	0.13	0.02	0.03	0.39	412	359	327	294
**Asia**												
South Asia	2.20	1.15	1.03	2.08	0.29	0.03	0.02	0.38	22 188	5787	3668	3685
Southeast Asia	1.34	1.09	1.05	1.61	0.10	0.03	0.03	0.28	2365	2063	1133	866
Western Asia	1.69	1.37	1.25	1.70	0.11	0.10	0.11	0.22	454	1423	637	249
**Latin America and the Caribbean**												
Caribbean	1.30	1.07	1.17	1.16	0.13	0.03	0.11	0.10	343	111	198	112
Central America	1.69	1.07	1.04	1.58	0.19	0.03	0.03	0.29	2155	564	228	864
South America	1.27	1.19	1.09	1.11	0.09	0.07	0.07	0.08	1963	2701	685	1250

Highlighted cells indicate the subgroup with the highest inequality within a region for each measure of inequality used. Subgroup categories are as follows: age (15–19, 20–24, 25–34 and 35–49 years), household wealth (five quintiles), residence (rural or urban) and parity (0 births or one or more births).

Moving further along the continuum of care, the dominant disparities in maternal healthcare are quite different than those for contraceptive services and are more consistent within and across geographical regions and the different inequality measures of magnitude and impact. Among live births where the mother received at least four or more ANC visits, the largest relative gaps in coverage were by household wealth quintile in every region, ranging from 1.14 in Central America to 3.53 in South Asia (ie, ANC coverage at the highest level in South Asia was 3.5 times that of coverage at the lowest level) (highlighted cells in table 2). Economic disparities also dominate in five regions when using a measure of the average absolute difference in coverage from the highest level in a subgroup. For the other regions, the urban–rural difference is larger for ANC coverage, ranging from six percentage points in Central America to 26 percentage points in Middle Africa. Yet reducing inequalities by economic status would result in the largest number of additional births where mothers had received ANC in every region compared with reductions in other subgroup inequalities.

**Table 2:** Inequality in the proportion of births to women aged 15–49 years who received four or more ANC visits by indicator, region and subgroup, 2017

	Relative measure (unweighted pairwise ratio)				Absolute measure (unweighted average absolute mean difference from highest subgroup category)				Population impact measure (number of additional births with 4+ANC visits if all were like the highest subgroup category (000 s))			
	Age	Wealth	Residence	Parity	Age	Wealth	Residence	Parity	Age	Wealth	Residence	Parity
**Africa**												
Eastern Africa	1.34	1.99	1.53	1.26	0.15	0.26	0.21	0.11	1875	3095	2283	1165
Middle Africa	1.08	1.69	1.64	1.25	0.03	0.23	0.26	0.11	140	996	912	488
Western Africa	1.32	3.09	1.84	1.19	0.07	0.36	0.33	0.09	558	4302	3136	1036
Northern Africa	1.17	1.73	1.23	1.27	0.06	0.24	0.14	0.17	223	1095	493	710
**Asia**												
South Asia	1.54	3.53	1.63	1.37	0.09	0.32	0.25	0.15	818	9841	6500	3817
Southeast Asia	1.08	1.50	1.16	1.09	0.04	0.15	0.13	0.07	168	1268	734	463
Western Asia	1.21	1.98	1.97	1.35	0.07	0.24	0.40	0.21	116	809	592	621
**Latin America and the Caribbean**												
Caribbean	1.18	1.26	1.25	1.11	0.05	0.12	0.18	0.08	14	65	50	32
Central America	1.07	1.14	1.06	1.03	0.03	0.05	0.06	0.03	38	143	56	61
South America	1.07	1.22	1.15	1.06	0.04	0.08	0.12	0.05	97	368	152	148

Highlighted cells indicate the subgroup with the highest inequality within a region for each measure of inequality used. Subgroup categories are as follows: age (15–19, 20–24, 25–34 and 35–49 years), household wealth (five quintiles), residence (rural or urban) and parity (one birth or two or more births).

ANC, antenatal care.

The patterns in inequalities are similar for delivery in a health facility, where gaps in coverage by household wealth quintile and urban–rural residence are among the highest across regions and different measures of inequality (table 3). The relative gap in coverage of delivery at a health facility is highest by household wealth quintile in six regions, by urban–rural residence in two regions (Western Asia and the Caribbean) and at similar levels of relative inequality in two regions (Central America and South America). The absolute mean difference in coverage of delivery at a health facility ranges from 20 to 43 percentage points in the four regions where household wealth is the subgroup with the largest level of inequality, and from 13 to 37 percentage points in the six regions where urban–rural residence has the largest level of inequality. As with ANC coverage, reducing inequalities by economic status would have the largest impact on additional births covered by delivery at a health facility (the one regional exception is Western Asia where bringing coverage in rural areas up to the level of urban areas would have a larger impact).

**Table 3:** Inequality in the proportion of births to women aged 15–49 years that were delivered in a health facility by indicator, region and subgroup, 2017

	Relative measure (unweighted pairwise ratio)				Absolute measure (unweighted average absolute mean difference from highest subgroup category)				Population impact measure (number of additional births delivered in a health facility if all were like the highest subgroup category (000 s))			
	Age	Wealth	Residence	Parity	Age	Wealth	Residence	Parity	Age	Wealth	Residence	Parity
**Africa**												
Eastern Africa	1.30	2.34	1.82	1.44	0.08	0.34	0.36	0.21	773	4009	3999	2266
Middle Africa	1.03	1.82	1.48	1.12	0.02	0.27	0.29	0.08	84	1203	1027	361
Western Africa	1.21	3.99	2.01	1.32	0.06	0.43	0.37	0.15	472	5152	3497	1677
Northern Africa	1.11	1.57	1.25	1.24	0.05	0.21	0.16	0.15	161	951	540	642
**Asia**												
South Asia	1.32	1.69	1.22	1.21	0.11	0.20	0.14	0.14	1173	6345	3728	3480
Southeast Asia	1.11	2.10	1.30	1.16	0.06	0.23	0.19	0.11	297	1988	1112	720
Western Asia	1.09	1.29	1.52	1.21	0.03	0.11	0.30	0.15	60	376	442	450
**Latin America and the Caribbean**												
Caribbean	1.69	1.55	1.74	1.24	0.19	0.22	0.37	0.15	76	114	101	59
Central America	1.04	1.19	1.19	1.06	0.02	0.06	0.16	0.06	41	170	161	116
South America	1.02	1.16	1.16	1.05	0.01	0.04	0.13	0.04	34	204	169	131

Highlighted cells indicate the subgroup with the highest inequality within a region for each measure of inequality used. Subgroup categories are as follows: age (15–19, 20–24, 25–34 and 35–49 years), household wealth (five quintiles), residence (rural or urban) and parity (one birth or two or more births).

## Conclusions

This analysis of inequalities across a continuum of reproductive health services, geographical regions, measures of inequality and key sociodemographic subgroups shows that not all inequalities in health coverage are ‘equal’. Dominant inequalities in contraceptive coverage are more varied, and include large disparities and impact by age group and parity, compared with maternal health services, where inequalities are consistently patterned and largest by economic status and urban–rural residence. Moving from contraceptive care to ANC and delivery is akin to moving from less urgent to more urgent reproductive health services and from more stigmatised to less stigmatised services. Contraceptive use by its very nature involves social barriers linked to women’s sexuality, especially social norms that restrict young women’s sexual activity to marriage and childbearing, in contrast to maternal health services where sexuality is less of a barrier to service use. These different patterns in inequality across services also refute the idea that adolescents are uniformly at a disadvantage with respect to reproductive health services. This is more the case for contraceptive coverage, but not so for maternal health services where financial and geographical barriers to access are strong.

Our summary picture of inequalities in reproductive health service coverage across developing regions has limitations. One limitation is that we examined subgroups separately, whereas in reality they intersect (eg, economic disparities within urban and rural areas or age-related disparities by stage in family formation).^15 16^ There are also relatively high but not complete correlations between some of the subgroups (eg, age and parity for contraceptive use or wealth and residence across all three health service outcomes; see online supplementary table 4). Another limitation is that service coverage tells a partial story of meeting population need for services. For example, a study of ANC in 10 countries showed a low quality of care, with missing components of routine care, even at relatively high levels of ANC coverage.^17^

Using multiple measures of inequality (relative, absolute and population impact) not only helps to show if there are consistent patterns in inequalities but also whether few or many different approaches are needed to reduce these inequalities and where resources could be prioritised to reach the largest number of people in need. Implications for programmes and policies include the types of approaches to prioritise to focus on the most disadvantaged people, such as voucher programmes or reduced user fees to address economic disparities, community sensitisation programmes to address age-related social barriers or expanding mobile outreach, community health workers or transportation and referral systems to address place-based disparities. While these findings highlight distinct patterns in inequalities across reproductive health services in developing regions, the design and implementation of policies and programmes to reduce inequalities are necessarily at the national and subnational levels. These issues are also pertinent for high-income countries. In the context of resource constraints, further information on the population impact of addressing different inequalities helps to quantify the tradeoffs of programme and policy efforts to reduce inequalities in health service coverage.
