# Supplementary material for: Not all inequalities are equal: differences in coverage across the continuum of reproductive health services
Source: BMJ Glob Health. 2019 Sep 3;4(5):e001695. doi: 10.1136/bmjgh-2019-001695 (PMC6730583; doi:10.1136/bmjgh-2019-001695)
Supplement: Supplementary data [file bmjgh-2019-001695supp001.pdf]

Supplementary materials

Table S1 Data sources used to estimate distributions of women aged 15–49 and births by contraceptive use, antenatal care and delivery at a facility by subgroup, countries in developing regions

Major area, region and country	Survey
**AFRICA**	
**Eastern Africa**	
Burundi	DHS 2010
Comoros	DHS 2012
Ethiopia	DHS 2011
Kenya	DHS 2014
Madagascar	DHS 2009
Malawi	DHS 2015
Mozambique	DHS 2011
Rwanda	DHS 2014
South Sudan	MICS 2010
Tanzania	DHS 2016
Uganda	DHS 2011
Zambia	DHS 2014
Zimbabwe	DHS 2015
**Middle Africa**	
Cameroon	DHS 2011
Central African Republic	MICS 2010
Chad	DHS 2014
Congo	DHS 2012
Dem. Rep. of the Congo	DHS 2014
Gabon	DHS 2012
Sao Tome and Principe	MICS 2014
**Western Africa**	
Benin	DHS 2012
Burkina Faso	DHS 2010
Côte d'Ivoire	DHS 2012
Gambia	DHS 2013
Ghana	DHS 2014
Guinea	DHS 2012
Guinea-Bissau	MICS 2014
Liberia	DHS 2013
Mali	DHS 2013
Mauritania	MICS 2011
Niger	DHS 2012
Nigeria	DHS 2013
Senegal	DHS 2014
Sierra Leone	DHS 2013

Togo	DHS 2014
**Northern Africa**	
Algeria	MICS 2012
Egypt	DHS 2014
Morocco	DHS 2003
Sudan	MICS 2014
Tunisia	MICS 2011
**ASIA**	
**Southern Asia**	
Bangladesh	DHS 2014
Bhutan	MICS 2010
India	NFHS 2016
Maldives	DHS 2009
Nepal	MICS 2014
Pakistan	DHS 2012
**Southeast Asia**	
Cambodia	DHS 2014
Indonesia	DHS 2012
Laos	MICS 2011
Philippines	DHS 2013
Thailand	MICS 2013
Timor-Leste	DHS 2009
Viet Nam	MICS 2010
**Western Asia**	
Armenia	DHS 2010
Azerbaijan	DHS 2006
Iraq	MICS 2011
Jordan	DHS 2012
State of Palestine	MICS 2014
Turkey	DHS 2008
Yemen	DHS 2013
**LATIN AMERICA AND THE CARIBBEAN**	
**Caribbean**	
Barbados	MICS 2012
Cuba	MICS 2014
Dominican Republic	MICS 2014
Haiti	DHS 2012
Saint Lucia	MICS 2012
**Central America**	
Belize	MICS 2011
Guatemala	DHS 2015
Honduras	DHS 2012
Mexico	MICS 2015

Nicaragua	ENDESA-RHS 2006
Panama	MICS 2013
**South America**	
Bolivia	DHS 2008
Brazil	PNDS 2006
Colombia	DHS 2010
Ecuador	ENDEMAIN-RHS 2004
Guyana	MICS 2014
Paraguay	ENDSSR-RHS 2008
Peru	DHS 2014

Notes: DHS=Demographic and Health Survey. ENDEMAIN=Encuesta Demográfica y de Salud Materna e Infantil. ENDESA=Encuesta Nicaragüense de Demografía y Salud. ENDSSR=Encuesta Nacional de Demografía y Salud Sexual y Reproductiva. MICS=Multiple Indicator Cluster Survey. NFHS=National Family Health Survey. RHS=Reproductive Health Survey.

Sources: Guttmacher Institute tabulations from surveys and survey reports. See Section 4 of Darroch JE, Adding It Up: Investing in Contraception and Newborn Health, 2017--Estimation Methodology, New York: Guttmacher Institute, 2017.

Table S2 Population-weighted percentage of women aged 15-49 and births for which survey data were available, according to region, 2017

	Number of countries	% women age 15-49 with survey data	% of births with survey data
Developing regions	148	66%	77%
Africa	55	90%	92%
Eastern Africa	18	95%	95%
Middle Africa	9	83%	81%
Southern Africa	5	9%	13%
Western Africa	16	100%	100%
Northern Africa	7	97%	98%
Asia	59	58%	67%
Eastern Asia	6	0%	0%
Central Asia	5	37%	41%
South Asia	9	92%	93%
Southeast Asia	11	85%	87%
Western Asia	18	67%	71%
Oceania	10	0%	0%
Latin America and the Caribbean	34	82%	82%
Caribbean	13	77%	81%
Central America	8	94%	95%
South America	13	78%	75%

Notes: Shading indicates subregions excluded from the analysis because relevant survey data were available for less than 50% of women of reproductive age in the subregion.

Table S3 Coverage of contraceptive and maternal health services by region and subgroup, 2017

% of women aged 15-49 years who have their need for family planning satisfied with modern methods														
	**Total**	**15-19**	**20-24**	**25-34**	**35-49**	**Poorest**	**Poorer**	**Middle**	**Richer**	**Richest**	**Rural**	**Urban**	**0 births**	**1+ births**
**Africa**														
Eastern Africa	59%	42%	60%	63%	57%	50%	55%	59%	61%	65%	55%	66%	48%	60%
Middle Africa	28%	27%	32%	27%	24%	15%	20%	24%	33%	37%	19%	37%	41%	24%
Western Africa	38%	33%	40%	40%	38%	22%	28%	35%	41%	52%	31%	47%	49%	36%
Northern Africa	71%	40%	66%	73%	72%	69%	69%	72%	72%	72%	69%	72%	33%	72%
**Asia**														
Southern Asia	71%	37%	50%	68%	81%	63%	70%	73%	73%	73%	70%	72%	35%	72%
Southeast Asia	72%	56%	69%	76%	72%	72%	74%	75%	72%	69%	74%	71%	46%	74%
Western Asia	53%	33%	47%	55%	53%	44%	49%	52%	56%	60%	45%	56%	32%	54%
**Latin America and the Caribbean**														
Caribbean	73%	61%	64%	73%	79%	70%	73%	73%	72%	75%	65%	76%	64%	75%
Central America	77%	50%	71%	75%	84%	74%	75%	76%	78%	79%	74%	77%	50%	80%
South America	81%	66%	78%	81%	84%	72%	79%	80%	83%	85%	75%	82%	74%	83%
**% births with 4 or more antenatal care visits**														
	**Total**	**15-19**	**20-24**	**25-34**	**35-49**	**Poorest**	**Poorer**	**Middle**	**Richer**	**Richest**	**Rural**	**Urban**	**1 birth**	**2+ births**
**Africa**														
Eastern Africa	44%	43%	43%	41%	57%	33%	37%	40%	48%	66%	39%	60%	52%	41%
Middle Africa	49%	47%	51%	47%	50%	45%	40%	45%	51%	67%	40%	66%	58%	46%
Western Africa	51%	41%	49%	55%	51%	26%	37%	52%	66%	81%	40%	73%	58%	49%
Northern Africa	68%	68%	72%	68%	61%	51%	60%	67%	76%	87%	62%	77%	80%	64%
**Asia**														
Southern Asia	47%	42%	49%	47%	32%	21%	39%	51%	61%	75%	40%	65%	58%	42%
Southeast Asia	84%	79%	83%	85%	82%	64%	81%	87%	92%	96%	78%	91%	88%	81%
Western Asia	66%	63%	67%	69%	57%	43%	58%	70%	75%	85%	41%	81%	82%	60%

**Latin America and the Caribbean**														
Caribbean	83%	85%	85%	83%	72%	74%	77%	84%	87%	93%	72%	90%	88%	79%
Central America	92%	91%	93%	93%	88%	85%	93%	95%	94%	97%	89%	94%	94%	91%
South America	86%	82%	86%	88%	84%	76%	85%	88%	91%	93%	77%	89%	89%	84%
**% of births delivered in a health facility**														
	**Total**	**15-19**	**20-24**	**25-34**	**35-49**	**Poorest**	**Poorer**	**Middle**	**Richer**	**Richest**	**Rural**	**Urban**	**1 birth**	**2+ births**
**Africa**														
Eastern Africa	52%	58%	54%	51%	44%	34%	43%	49%	61%	81%	44%	81%	68%	47%
Middle Africa	70%	71%	70%	69%	72%	51%	62%	71%	81%	93%	60%	90%	77%	68%
Western Africa	49%	44%	49%	53%	47%	22%	33%	50%	67%	86%	37%	74%	61%	47%
Northern Africa	68%	64%	71%	67%	66%	54%	60%	68%	75%	85%	62%	77%	79%	64%
**Asia**														
Southern Asia	71%	63%	74%	71%	56%	53%	66%	74%	81%	89%	67%	81%	81%	67%
Southeast Asia	74%	69%	73%	77%	69%	44%	69%	78%	88%	93%	65%	85%	81%	70%
Western Asia	76%	75%	75%	77%	71%	66%	73%	77%	81%	85%	57%	87%	87%	72%
**Latin America and the Caribbean**														
Caribbean	72%	84%	75%	71%	50%	58%	64%	73%	76%	90%	50%	87%	81%	65%
Central America	92%	93%	93%	92%	89%	81%	92%	95%	97%	97%	81%	97%	96%	90%
South America	92%	92%	92%	92%	90%	82%	92%	95%	95%	95%	81%	95%	94%	90%

Table S4 Pearson correlation coefficients for subgroup inequalities measured using the unweighted average absolute mean difference from highest subgroup category

**% of women aged 15-49 years who have their need for family planning satisfied with modern methods**				
	Age	Wealth	Residence	Parity
Age	1			
Wealth	-0.66	1		
Residence	-0.70	0.85	1	
Parity	0.65	-0.51	-0.71	1
**% births with 4 or more antenatal care visits**				
	Age	Wealth	Residence	Parity
Age	1			
Wealth	0.56	1		
Residence	0.33	0.74	1	
Parity	0.37	0.62	0.68	1
**Facility delivery**				
	Age	Wealth	Residence	Parity
Age	1			
Wealth	0.31	1		
Residence	0.40	0.67	1	
Parity	0.54	0.64	0.60	1
